# Functional Annotation of Alzheimer’s Disease-Associated SNPs Based on Three-Dimensional Chromatin Structure

**DOI:** 10.3390/ijms27188396

**Published:** 2026-09-20

**Authors:** Haitao Li, Zhen Wang, Xin Liu, Xiao Sun

**Affiliations:** 1 Key Laboratory of Intelligent Computing and Signal Processing of Ministry of Education, School of Artificial Intelligence, Anhui University, 111 Jiulong Road, Economic and Technological Development Zone, Hefei 230601, China; leehightall@163.com (H.L.); w125221265@stu.ahu.edu.cn (Z.W.); 18712039763@163.com (X.L.); 2 State Key Laboratory of Bioelectronics, School of Biological Science and Medical Engineering, Southeast University, Nanjing 210096, China

**Keywords:** Alzheimer’s disease, genome-wide association study, non-coding variants, three-dimensional chromatin structure, Hi-C, promoter capture Hi-C, candidate regulatory target genes

## Abstract

Genome-wide association studies (GWASs) have identified numerous loci associated with Alzheimer’s disease (AD), yet the effector genes and regulatory mechanisms underlying many of these associations remain unresolved. This challenge is particularly pronounced for non-coding variants, whose regulatory effects may extend over long genomic distances and cannot be reliably inferred from the nearest gene alone. Here, we developed an integrative computational framework that incorporates three-dimensional chromatin interactions, expression quantitative trait loci (eQTL), and sequence-level regulatory annotations to identify candidate effector genes at AD-associated loci. AD-associated lead single-nucleotide polymorphisms (SNPs) and variants in strong linkage disequilibrium were mapped to enhancer elements and promoter-interacting regions using publicly available Hi-C and promoter capture Hi-C data from the hippocampus and cerebral cortex. Hi-C-derived enhancer–promoter relationships were inferred using PSYCHIC, whereas significant promoter-centered interactions from promoter capture Hi-C were used to connect variant-containing regions with candidate effector genes. The resulting SNP–gene relationships were further evaluated using brain-relevant eQTL evidence and predicted allele-dependent alterations in transcription factor binding motifs. This integrative analysis identified 608 unique candidate regulatory target genes supported by spatial chromatin contacts and complementary regulatory evidence, including genes not necessarily assigned by conventional nearest-gene annotation. Functional enrichment analysis indicated that the prioritized genes were involved in biological processes relevant to AD pathophysiology. Detailed analyses of three representative SNP–gene pairs, rs2373115–*NARS2*, rs6656401–*CR1*, and rs3776011–*ACSL6*, further illustrated how chromatin interaction, expression-associated, and sequence-level evidence can be combined to formulate locus-specific regulatory hypotheses. These findings represent computationally inferred and associative regulatory relationships rather than experimentally validated causal effects. They provide a structured framework for refining post-GWAS interpretation of non-coding AD risk loci and prioritizing candidate targets for further experimental validation.

## 1. Introduction

Genome-wide association studies (GWASs) have been successful in identifying a large number of genetic variants associated with common diseases and medically important traits in large cohorts [1]. Currently, there are sufficient GWASs to justify the existence of specialized databases, such as the GWAS Catalog [2]. However, despite this wealth of data, GWAS findings have not been successfully translated into many effective treatments. The precise pathogenic mechanisms of the vast majority of these associated loci remain unknown. It has been found that most GWAS-significant single-nucleotide polymorphisms (SNPs) reside in non-coding regions and may perturb gene expression through multiple functional mechanisms rather than alter protein function [3].

Functional annotations of non-coding SNPs mapped by GWASs are still difficult and challenging. This difficulty arises primarily from the complex linkage disequilibrium (LD) between SNPs and causative genes and, secondarily, from sampling errors in test statistics [4]. It is intuitively assumed that genes located in closest physical proximity to top-associated variants represent the most likely target genes. Unfortunately, this hypothesis remains insufficiently validated because of the lack of comprehensive follow-up functional studies of GWAS findings [5]. Moreover, recent studies have provided examples suggesting that the actual target genes may differ from the nearest genes [4,6]. One well-documented example involves two independent obesity-linked SNPs located within an intron of the FTO gene: these variants have been found not to regulate FTO itself but instead modulate IRX3 in brain tissue and both IRX3 and IRX5 in adipocytes [6,7]. This FTO obesity locus exemplifies the highly complex, cell-type-specific mechanism by which non-coding variants participate in disease development. Nevertheless, thoroughly annotated cases of this type remain uncommon, and the systematic mapping of GWAS-identified SNPs to their corresponding regulatory targets has not yet been fully established, particularly in the setting of complex neuropsychiatric disorders such as Alzheimer’s disease (AD) [8].

Late-onset AD, the most prevalent form of dementia globally, affects more than 44 million people worldwide and presents clinically with progressive memory impairment, cognitive deterioration, and neurodegeneration [9]. At the pathophysiological level, AD is marked by synaptic loss, which is primarily associated with the deposition of β-amyloid peptides in extracellular plaques and the intracellular aggregation of abnormally hyperphosphorylated microtubule-associated protein tau, with these pathological changes being most prominent in the hippocampus and cerebral cortex [10]. Recent progress in the genomics of late-onset AD, alongside high-throughput sequencing technologies and large-scale GWASs, has enabled the investigation of not only major causal genes underlying the disease but also multiple low-frequency genetic loci that appear to exert substantial effects on AD susceptibility. Even though these approaches have uncovered novel AD-associated genes, GWAS-derived findings have yet to be integrated into a genetic epidemiology framework for individualized risk prediction [11]. A key bottleneck hindering this translational process is the identification of bona fide causal genes from GWAS outputs; such genes can later serve as therapeutic targets and thus support the development of novel treatments. This disconnect between genetic research findings and their translational applications is primarily attributable to the challenges associated with the functional interpretation of non-coding variants [12].

To narrow this gap, a number of experimental techniques have been developed, such as expression quantitative trait locus (eQTL) analysis [4]. When the expression level of a given gene is modulated by a genetic variant, variations in transcript abundance can be detected across individuals harboring distinct genotypes of that variant (e.g., the AA, Aa, and aa genotype groups). This genotypic effect on gene expression follows a pattern analogous to experimental overexpression and knockdown assays: specifically, the AA genotype aligns with overexpression conditions, Aa matches baseline wild-type expression levels, and aa corresponds to transcriptional suppression. However, eQTL analysis is primarily aimed at evaluating potential cis associations rather than establishing a causal association between SNPs and the expression of their target genes. Establishing a definitive causal association requires additional supporting evidence, as eQTL analysis on its own provides inadequate support for such causal claims. Furthermore, there exists a common tendency to attribute causal roles to variants even when alternative causal explanations remain plausible, and rigorous empirical validation is required to establish unambiguous causal effects [13].

A growing number of studies have found that physical chromatin interactions may mediate the effects of cis-acting regulatory variants, including eQTLs, on gene expression [8]. Thanks to the development of high-throughput sequencing techniques, our knowledge of the three-dimensional (3D) chromatin structure of the genome has greatly increased in recent decades [14,15]. Among these technologies, chromosome conformation capture (3C) [16] and its high-throughput derivatives have been the most prominent ones. High-throughput chromosome conformation capture (Hi-C) is the most comprehensive and high-throughput derivative, allowing us to quantitatively evaluate contact frequencies between almost any pair of genomic loci [17]. For research inquiries focused specifically on interactions between promoters and regulatory elements, or between targeted genomic loci such as SNPs, capture Hi-C represents a more appropriate methodological approach [18,19]. Promoter capture Hi-C (PCHi-C), a targeted derivative of capture Hi-C, is engineered to enrich for interactions anchored to a predefined set of annotated gene promoters [19,20]. By directly enriching for ligation products derived from the selected target sequences, this approach achieves higher resolution across promoter regions. Previous epigenomic annotations and analyses of the noncoding regions revealed the importance of a three-dimensional (3D) chromatin structure in gene regulation and disease [21,22,23]. For example, Meng et al. dysregulated enhancers are closely associated with neurodegenerative diseases like Alzheimer’s disease, and Hi-C datasets have been used to link Alzheimer’s risk factors and epigenetic marks to target genes, revealing long-distance promoter–enhancer interactions in known Alzheimer’s risk genes [24]. Patel et al. reveals that in AD, epigenetic dysregulation involving the master genome architecture protein CTCF leads to significant alterations in the 3D chromatin organization, particularly affecting genes related to synaptic organization and cell adhesion, possibly contributing to the reduced expression of these genes and implicating changes in histone modification as a potential mechanism [25].

Several computational strategies have been developed to interpret non-coding GWAS associations by linking variants to candidate target genes. For example, FUMA and INFERNO integrate positional and functional genomic annotations with eQTL and regulatory information; H-MAGMA incorporates brain chromatin-interaction profiles into gene-level GWAS analysis; S2G/cS2G frameworks integrate multiple SNP-to-gene linking strategies; and Activity-by-Contact (ABC)-based approaches predict cell-type-specific enhancer–gene relationships by combining regulatory activity with chromatin contact. In Alzheimer’s disease, Lutz et al. [26] further demonstrated the value of integrating brain chromatin-state annotations with enhancer–promoter interaction information for interpreting selected LOAD GWAS loci. Collectively, these studies establish that integration of functional genomic and chromatin-interaction information is an important component of post-GWAS interpretation. The present study does not aim to introduce a new statistical SNP-to-gene algorithm; rather, we systematically combine complementary brain Hi-C and promoter capture Hi-C information to map AD-associated variants in enhancers and promoter-interacting regions to candidate regulatory target genes and to generate locus-level hypotheses for subsequent functional investigation.

**Figure 1:**
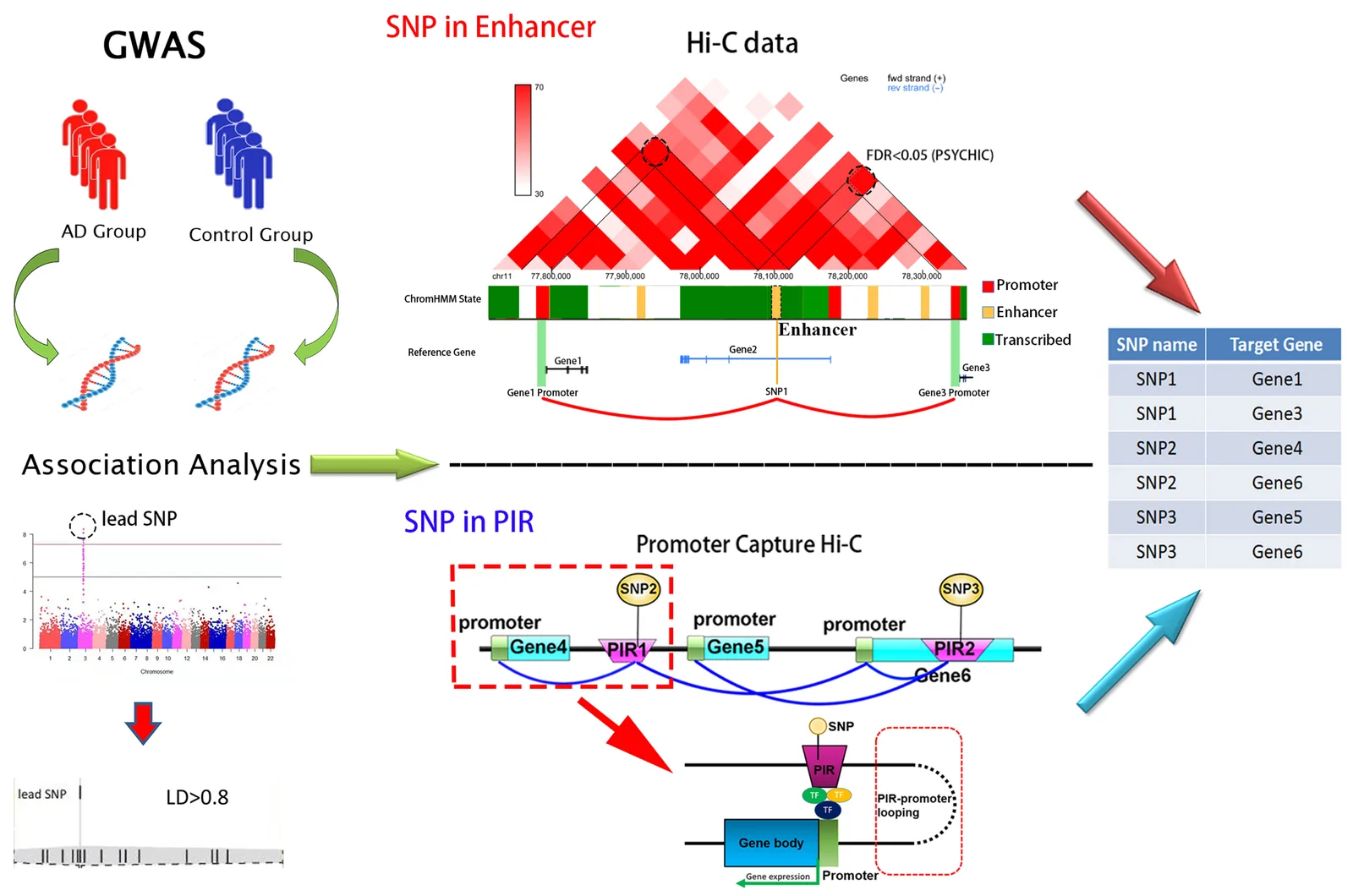
Our strategy for functional annotation of AD-associated SNPs. Following AD GWAS analysis, lead SNPs were downloaded from the GWAS Catalog database. AD-associated SNPs were identified at linkage disequilibrium threshold of 0.8 across all AD lead SNPs. To elucidate how the noncoding AD SNPs within enhancers and PIRs affect the gene expression levels to predispose AD development, chromatin–chromatin interactions are identified by Hi-C and PCHi-C experiments from brain tissues. PSYCHIC algorithm was performed to predict corresponding candidate effector genes when SNPs are located in enhancer regions via the Hi-C experiment, while significant long-range PIR–target genes promoter interactions were chosen to identify the candidate effector genes corresponding to the SNP via the PCHi-C experiment. Finally, a list of candidate effector genes was generated. Arrows indicate the direction of the analytical workflow and regulatory mapping. Different colors distinguish genomic elements and analytical components; shading in the Hi-C map reflects chromatin contact intensity. Dashed lines outline regulatory regions of interest.

In this purely in silico study, we leveraged 3D chromatin structure information, including Hi-C and PCHi-C data from two brain tissues (hippocampus and cerebral cortex), to functionally annotate AD-associated SNPs. These two regions of the brain were involved in functions affected by Alzheimer’s disease [27]. We focused on the functional annotation of AD-associated SNPs in enhancer element regions or/and promoter-interacting regions (PIRs). In brief, the PSYCHIC algorithm [28] was utilized to identify enhancer–target gene promoter pairs from Hi-C experiments in brain tissues [29] and predict corresponding candidate effector genes when SNPs are located in enhancer regions within these pairs. Meanwhile, significant long-range PIR–target gene promoter interactions were selected from PCHi-C data [30], and the candidate effector genes corresponding to the SNPs were identified in these interactions. Our strategy can identify candidate effector genes corresponding to the promoters that interact with AD-associated SNPs in enhancer regions or/and PIRs using chromosome conformation capture techniques, and a targeted list of candidate AD genes was generated, feasible for post-GWAS investigations of causality. Additionally, we explored the impact of the identified SNPs on the expression levels of candidate effector genes by integrating eQTL data from the GTEx database. Furthermore, to delve into the potential regulatory mechanisms linking SNPs to candidate effector genes, we evaluated their effects on transcription factor binding sites using information from the JASPAR database. Based on existing research, we inferred changes in chromatin interactions that may influence gene expression regulation, thus exploring their relationship with disease risk. The overall analytical framework is illustrated in Figure 1.

## 2. Results

### 2.1. Identification of Candidate Effector Genes Associated with AD-Associated SNPs

To investigate the potential regulatory mechanisms underlying AD-associated variants, we integrated SNP annotation with three-dimensional chromatin interaction information to identify candidate effector genes. Following LD-based expansion of AD-associated lead SNPs, 6164 unique variants were obtained (Supplementary File S1), among which 608 putative candidate effector genes were assigned based on chromatin interaction-mediated regulatory relationships (Supplementary File S2).

**Figure 2:**
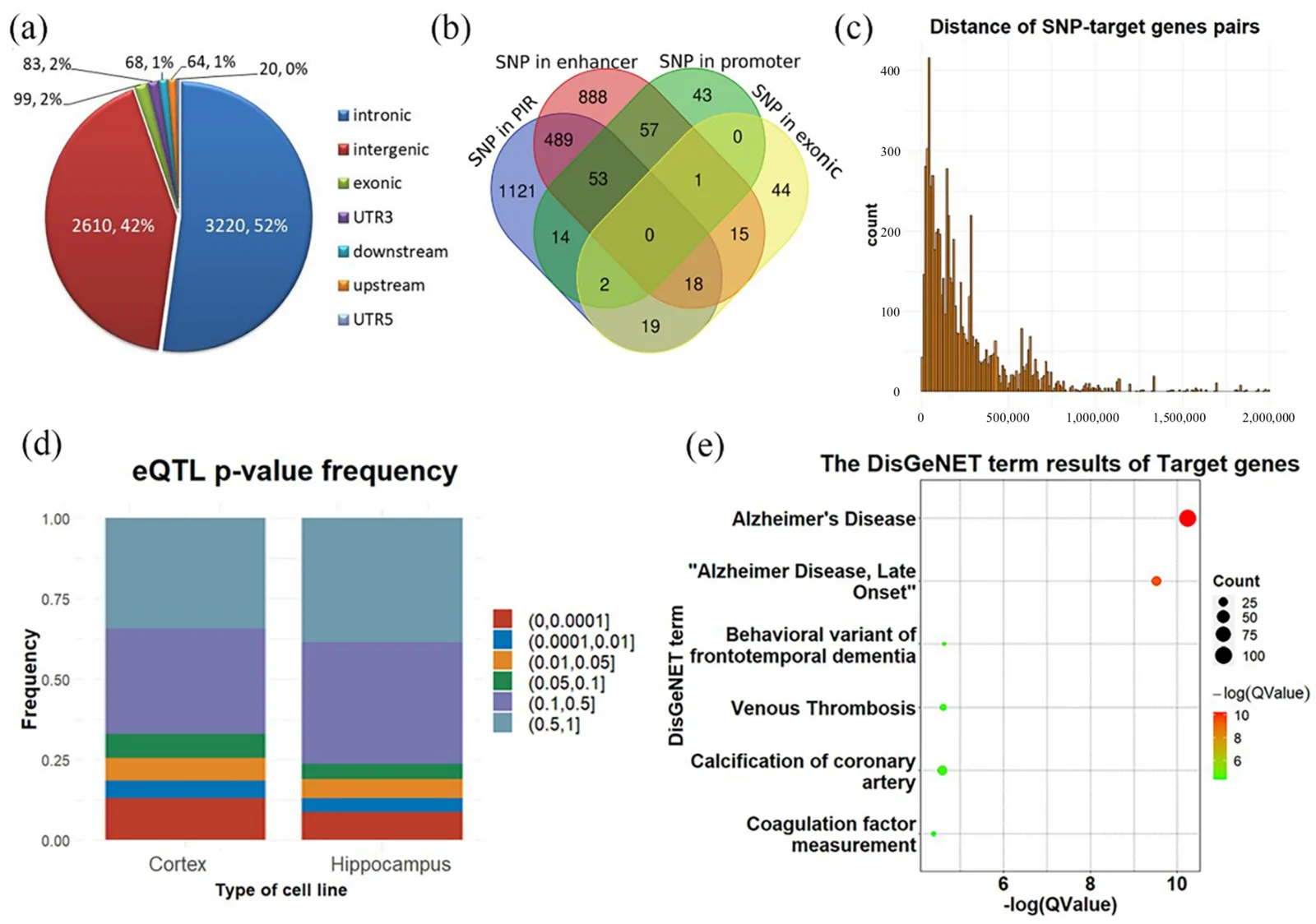
The general information of AD-associated SNPs and their candidate effector genes. (**a**) Pie chart depicting distribution of the AD-associated SNPs with ANNOVA according to genomic location. (**b**) Venn plot showing the overlaps of the regions where the functional annotation AD SNPs are located based on our strategy. (**c**) the distance between SNPs and the corresponding TSSs of candidate effector genes. (**d**) Percentage stacked bar chart displaying the distribution of eQTLs p-value of SNP–candidate effector genes pairs identified by our method from GTEx project in brain cortex and hippocampus. (**e**) The bubble diagram of diseases enrichment analyses of the putative candidate effector genes. In the bubble diagram, dot sizes represent counts of enriched target genes, and dot colors represent negative Log10 (*FDR* values).

The genomic distribution of AD-associated variants was first examined to characterize their regulatory properties. As shown in Figure 2a, more than 95% of AD-associated SNPs were located in noncoding regions, whereas only a small proportion were located within coding exons. These findings indicate that most AD-associated variants may contribute to disease susceptibility through regulatory mechanisms rather than through direct alteration of protein sequences.

To further characterize the regulatory relationships between identified SNPs and their candidate effector genes, we analyzed the genomic distance between SNP loci and transcription start sites (TSSs) of assigned genes. As shown in Figure 2b, AD-associated SNPs were distributed across enhancer regions and promoter-interacting regions, indicating that multiple types of regulatory elements contribute to AD-associated gene regulation. The distance analysis demonstrated that the median distance between SNPs and candidate effector genes was 158,223 bp, and 85.7% of SNP–gene interactions occurred within a range of 500 kb (Figure 2c). These results suggest that chromatin interaction-based annotation can identify distal regulatory relationships that may be missed by conventional nearest-gene assignment.

To evaluate whether the predicted SNP–gene relationships were associated with transcriptional regulation, we integrated brain tissue eQTL datasets from the GTEx database. Significant SNP–target gene associations were observed in both cortical and hippocampal tissues. Approximately 25% of predicted SNP–gene pairs showed nominal eQTL associations in cortical tissues, while approximately 20% showed significant associations in hippocampal tissues (Figure 2d). Furthermore, highly significant eQTL associations (p<0.01) were detected for 16% and 11% of SNP–gene pairs in cortical and hippocampal tissues, respectively. These results provide independent evidence supporting the regulatory potential of the identified SNP–gene interactions.

Recently, Mathys et al. conducted single-cell sequencing to study gene expression within each individual cell from AD patients and control samples [31]. They compared gene expression differences in various cell types between AD patient brains and control brains, identifying 1031 uniquely differentially expressed genes. Among these, 84 genes overlapped with the 608 genes we inferred. We used hypergeometric testing to examine the significance of the enrichment of inferred target genes among differentially expressed genes in AD and normal samples. The results suggest that our inferred candidate effector genes may be regulated by AD-associated SNPs in non-coding regions (*p*-value = 6.85 $\times \;10^{-13}$).

Subsequently, we conducted enrichment analysis using the Enrichr [32] gene-disease association network (DisGeNET) [33] and dbGaP [34] datasets to investigate the associations between the 608 inferred candidate effector genes and potential diseases. We used an *FDR* threshold of less than 0.05 to select significantly enriched diseases related to the target genes. The disease enrichment analysis from dbGaP indicated only ‘Alzheimer’s Disease,’ while DisGeNET analysis revealed significant enrichment of candidate effector genes associated with AD and neuro-related diseases (Figure 2e). This indicates a close association between the inferred candidate effector genes identified using our strategy and Alzheimer’s disease.

### 2.2. The Significant Pathway Crosstalk Network Based on Candidate Effector Genes

Conventional pathway enrichment identifies biological pathways that are individually overrepresented among candidate genes but does not explicitly describe how these enriched pathways are related to one another. We therefore performed a pathway crosstalk analysis to quantify gene sharing among significantly enriched pathways and to identify higher-order functional modules. Fifteen pathways were significantly enriched using ToppGene with *FDR* < 0.05 (Table 1).

**Figure 3:**
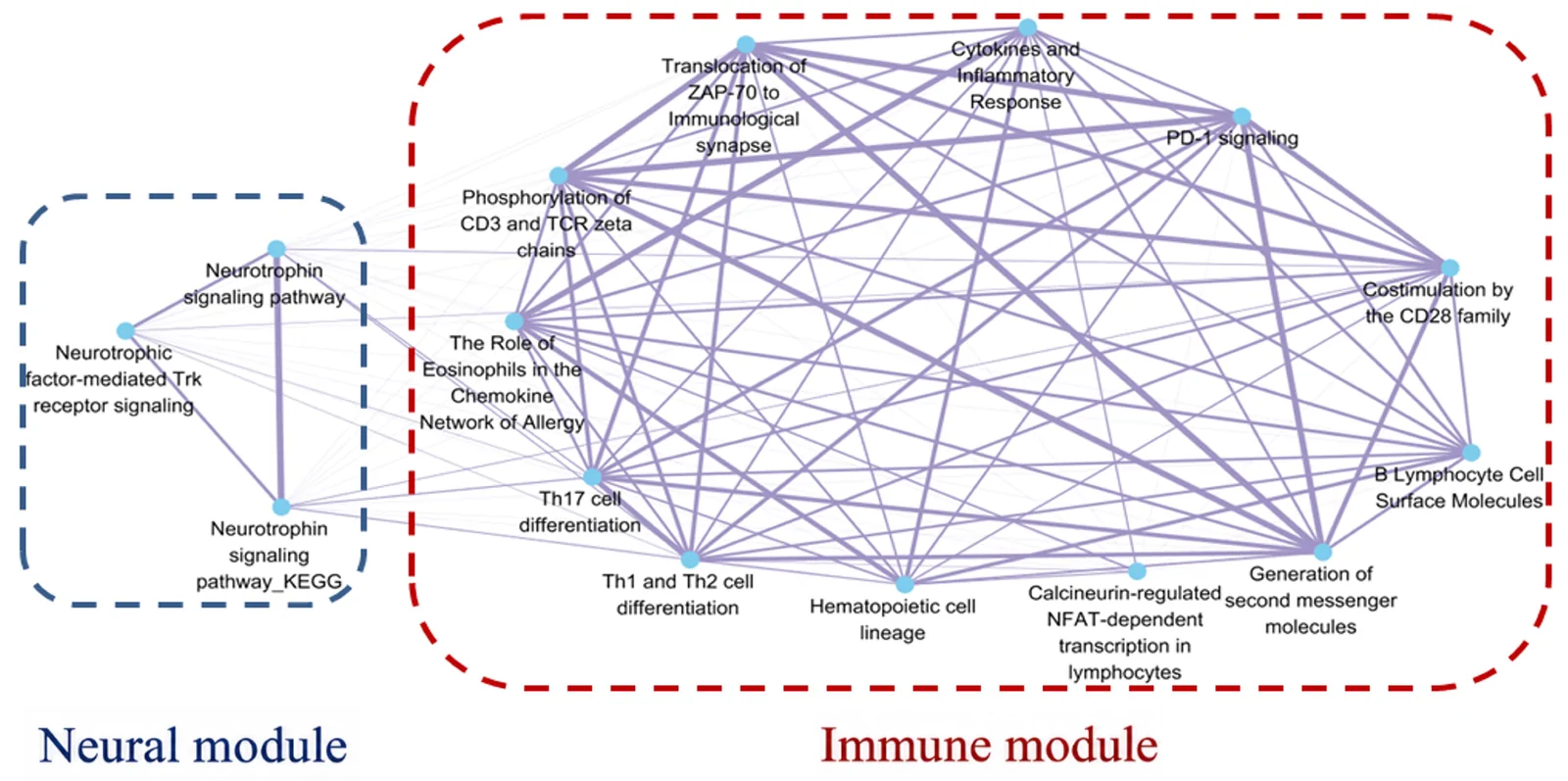
The significant pathway crosstalk network. Each vertex is a biological pathway. Each line is crosstalk among each pathway pair. Width of one edge indicates the overlap value with the crosstalk level of a given pathway pair. These pathways could be divided into two major modules by the spectral clustering algorithm. One is neural module (the left module), the other is immune module (the right module). Edge width is proportional to the overlap value between paired pathways, with thicker lines indicating a higher degree of gene sharing. Full overlap values for all pathway pairs are provided in Supplementary File S4.

We utilized the method described in the Section 4 to construct a network of pathways enriched with significantly associated target genes. The crosstalk network is built by calculating the overlap of candidate effector genes between significantly associated pathways. The higher the biological similarity and functional overlap between pathways, the stronger the connection between them, resulting in denser relationships in the network, as depicted in Figure 3. Based on the significantly enriched pathways identified in the constructed network, these pathways could be divided into two major modules using spectral clustering, namely, the neural module and the immune module.

The first module primarily consisted of biological pathways related to neural functions, including ‘Neurotransmitter Signaling Pathway’ and ‘Neurotrophin-mediated Trk Receptor Signaling.’ The second module was predominantly composed of biological pathways closely associated with immune responses, such as ‘ZAP-70 Transfer to Immunological Synapse,’ ‘PD-1 Signaling Pathway,’ ‘Cytokines and Inflammatory Response,’ and ‘Phosphorylation of CD3 and TCR ζ Chains,’ among others. These immune-related pathways in the module are strongly associated with the onset and development of Alzheimer’s disease (AD). For instance, recent research has demonstrated that the PD-1 signaling pathway can regulate the expression of the IL-17 cytokine in helper T cells 17 (Th17), suppress CD3-TCR signal transduction, and terminate ZAP-70 and PI3K phosphorylation, consequently affecting downstream signaling pathways including PI3K-AKT and RAS-ERK. This immunomodulatory role plays a significant role in central nervous system diseases [31]. Disturbance of the PD-1 signaling pathway can lead to abnormalities in downstream signaling pathways, potentially triggering AD. Through the crosstalk network we constructed, we can consolidate the relevant signaling pathways that affect AD, facilitating targeted research into their regulation, interactions, and connections with the pathogenesis of AD.

### 2.3. Examples of AD Risk Variant SNP Regulates Gene Expression

To validate the effectiveness of the annotation strategy, we selected two groups of “SNP-target gene pairs” with high interaction confidence in the experimental results for detailed functional annotation. We specifically analyzed the process of SNP-regulated gene expression and how it influences the risk of AD.

In some independent studies, GWAS confirmed the involvement of the rs2373115 (C>A) variant in AD risk [35,36]. Rs2373115 is located in the intron of *GAB2* and within an enhancer. Liu et al. found it reduced *NARS2* expression in AD cases compared with controls using a large-scale AD case–control expression dataset [37].

**Figure 4:**
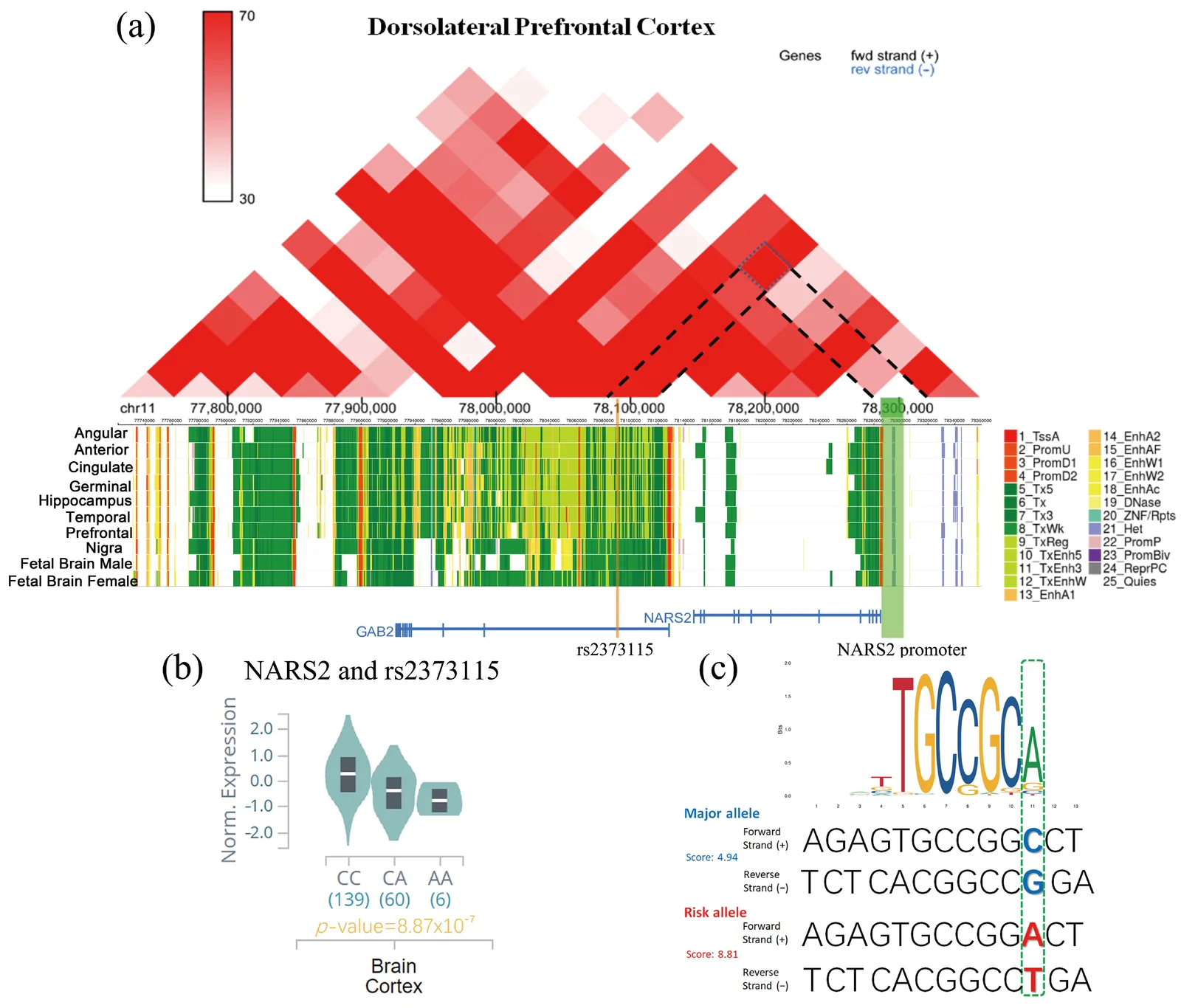
SNP rs2373115 is associated with *NARS2* expression. (**a**) Hi-C experiment from brain cortex showed significant chromatin interaction between rs2373115 and promoters of *NARS2* by PSYCHIC. We used the 3D Genome Browser to visualize Hi-C data, and gene structure and chromHMM track defined via 25-state ChromHMM model for ten brain tissues was shown using WashU Epigenome Browser. Color intensity in the Hi-C map represents chromatin contact intensity. ChromHMM track colors correspond to the 25 chromatin states in the legend. The dashed box marks the region of interest. (**b**) Violin plot showing the effect of the eQTL rs2373115 on *NARS2* expression in brain cortex; (**c**) Predicted effect of rs2373115 on the ZFP57-binding motif based on JASPAR analysis. The top sequence logo shows nucleotide preferences within the motif: letter height represents preference degree, and different colors distinguish nucleotides. Green dashed boxes mark rs2373115 position, and colored bases indicate alternative alleles for comparison.

In this analysis, the Hi-C experiment from the brain cortex showed a significant chromatin interaction between rs2373115 and the *NARS2* promoters (*FDR*-value = 2.1 $\times \;10^{-10}$) (Figure 4a), and the rs2373115 A allele was significantly associated with decreased *NARS2* expression in the brain cortex (*p*-value = 8.87 $\times \;10^{-7}$), as examined in the GTEx V8 dataset (Figure 4b). However, the relatively small sample size of the AA genotype subgroup (*n* = 6) may limit the precision of the genotype-specific expression estimate, and this result should therefore be interpreted with caution.

Another example of an AD SNP regulating gene expression is rs6656401. Large-scale GWAS analyses have uncovered multiple novel genetic risk factors for AD; specifically, the variant rs6656401 within the *CR1* locus has been identified by two independent research teams [38,39]. Positioned in the intronic region of the *CR1* gene, rs6656401 does not alter the amino acid sequence or protein architecture of its target gene *CR1*. A growing number of studies have confirmed that dysregulation of *CR1* affects inflammation and amyloid accumulation, which significantly increases the risk of AD [40,41].

**Figure 5:**
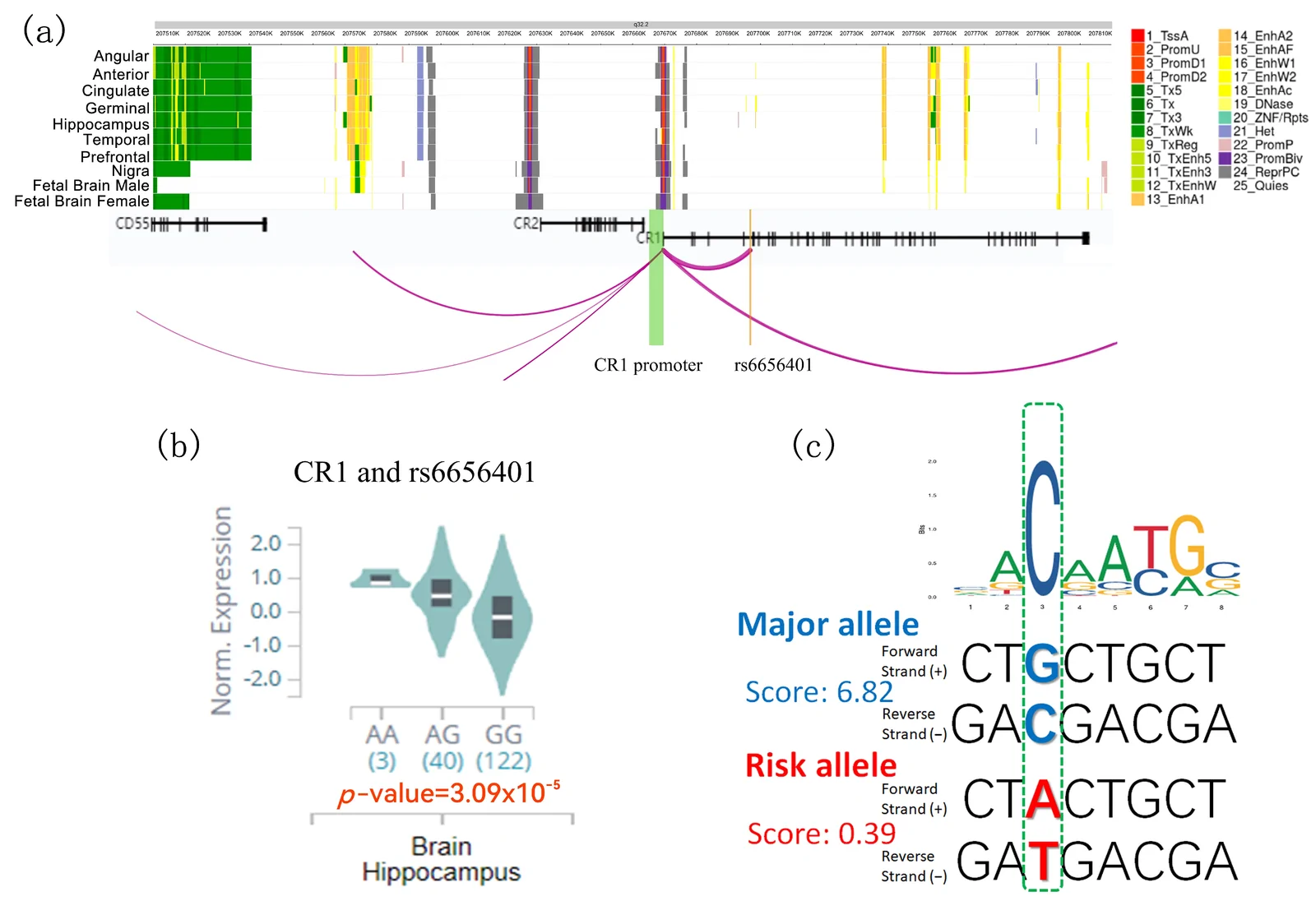
SNP rs6656401 is associated with *CR1* expression. (**a**) Rs6656401 in PIRs interacts with the *CR1* promoter from the PCHi-C experiment in brain hippocampus. Ellipse width is proportional to the interaction intensity of *CR1* promoter and PIR. (**b**) Violin plot showing the effect of the eQTL rs6656401 on *CR1* expression in brain hippocampus. (**c**) rs6656401 is located within a putative BRG1-binding region, suggesting that the variant may affect BRG1 binding at this locus. The top sequence logo shows nucleotide preferences of the BRG1-binding motif: letter height indicates the degree of nucleotide preference at each position, and different colors distinguish the four nucleotide bases. The green dashed box marks the position of rs6656401, and the colored bases represent the alternative alleles used for comparison.

In our analysis, a significant chromatin–chromatin interaction was identified by PCHi-C in the hippocampus between the *CR1* promoter and the PIR where the SNP is located (Figure 5a). The eQTL data from the GTEx project indicated that the rs6656401 A allele was significantly associated with increased *CR1* expression in hippocampal tissue (Figure 5b). The relatively small sample size of the AA genotype subgroup (*n* = 3) may further limit the precision of the genotype-specific expression estimate, and the observed association should therefore be interpreted with caution.

In addition, the genetic variation rs6656401 is located within a putative TF binding site for BRG1, identified in a single cell line using ChIP-Seq data. Rs6656401 could affect the binding of BRG1 to the promoter [42]. BRG1 is a critical modulator of transcriptional regulation in cellular processes [43]. In line with this, several independent investigations have indicated that BRG1 may play a regulatory role in shaping the structural organization and functional state of chromatin at TAD boundaries through modulating the functional activity and recruitment of CTCF and DNA topoisomerase complexes [44]. Hence, TF BRG1 plays an essential role in mediating the establishment and/or maintenance of long-range DNA interactions [45].

### 2.4. Regulatory Evidence Linking rs3776011 to ACSL6

Based on the analysis of 3D genomic data, the SNP rs3776011 was linked to six candidate target genes based on 3D genomic annotation. Additionally, the promoter region of the *ACSL6* gene is regulated by thirteen SNPs. Genomic details for all these variants and candidate genes are provided in Tables S1 and S2 of Supplementary Materials S5. This locus is used as a representative example to illustrate how brain 3D chromatin information can refine the functional interpretation of distal non-coding AD-associated variants and provide new insights into the molecular mechanisms of the disease.

rs3776011 (chr5: 130868840, C>T, GRCh37) is located in the intronic region of RAPGEF6 and within active enhancer elements of brain tissues. It is in high LD ($r^{2}$ = 0.89 and D’ = 1) with the statistically significant AD-associated SNP rs6890695 [46] (chr5: 130888424, C>T, GRCh37). Variants across the broader 5q31.1 genomic interval, including association signals near both RAPGEF6 and *ACSL6*, have been previously reported in AD genetic and functional genomic studies [47]. In the linear genome, rs3776011 is approximately 478 kb away from the transcription start site of *ACSL6*.

**Figure 6:**
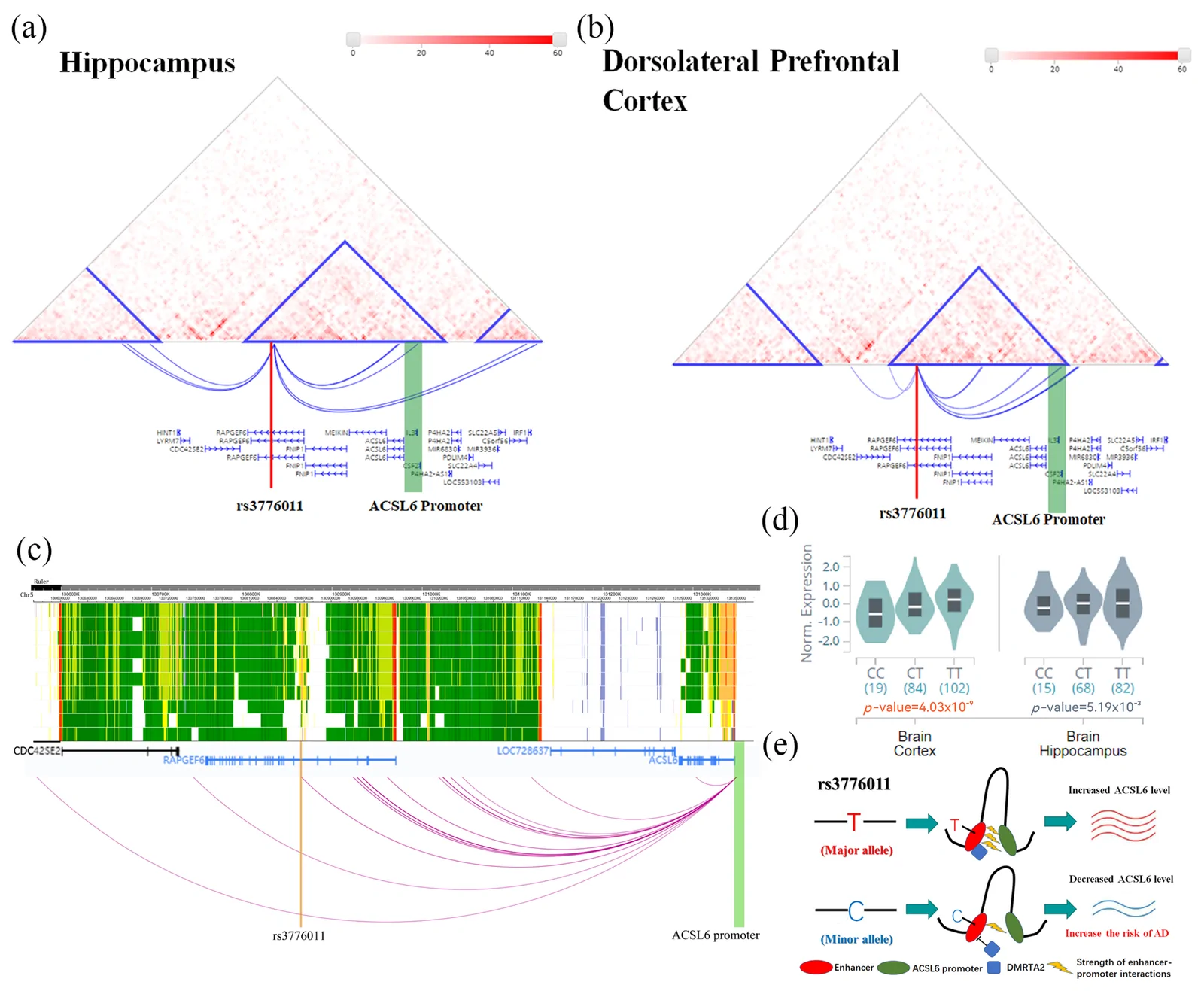
Multi-layer regulatory evidence linking rs3776011 to *ACSL6*. (**a**,**b**) Brain Hi-C maps showing the 3D genomic organization surrounding rs3776011 in the hippocampus (**a**) and brain cortex (**b**). Hi-C maps are shown at 40 kb resolution, with color intensity indicating chromatin interaction strength. Blue triangles indicate putative TADs called by TopDom. (**c**) PCHi-C evidence showing spatial interaction between the promoter-interacting region containing rs3776011 and the *ACSL6* promoter in brain cortex; arcs represent chromatin interactions. (**d**) GTEx v8 brain eQTL associations between rs3776011 genotype and *ACSL6* expression in cortex and hippocampus. (**e**) Proposed regulatory model integrating chromatin-contact, eQTL, and sequence-based motif evidence. JASPAR analysis predicts an allele-dependent difference in a putative DMRTA2-binding motif; this prediction does not establish allele-specific TF occupancy or altered enhancer–promoter interaction strength. In the model, red marks enhancers, green marks the *ACSL6* promoter, and blue marks DMRTF2. Dashed arrows indicate hypothesized regulatory relationships requiring experimental validation.

As shown in Figure 6a–c, despite the long distance between rs3776011 and the *ACSL6* promoter, SNP significantly interacts with its target gene over a long genomic distance based on 3D chromatin structure revealed by Hi-C and PCHi-C experiments. Meanwhile, the rs3776011 T allele was significantly associated with increased *ACSL6* expression in the brain cortex (*p*-value = $4.03\times 10^{-9}$) and hippocampus (*p*-value = $5.19\times 10^{-3}$), as examined in the GTEx V8 dataset.

Based on a meta-analysis of public gene expression data for neurodegenerative diseases (including AD) to identify a common transcriptional signature of neurodegeneration [48], *ACSL6* was one of the down-regulated genes identified in the study. This suggests that reduced expression of *ACSL6* may contribute to the development of AD. Recently, a growing number of biological experiments have also supported this observation [49,50]. *ACSL6* expressed in mature neurons is a major contributor to the omega-3 fatty acid docosahexaenoic acid (DHA) in the brain. Brain DHA content is inversely correlated with the risk of age-related neurological diseases, disorders, and cognitive decline [51]. Low levels of DHA and DHA-derived lipid mediators are associated with the pathophysiology of several diseases, including AD and Parkinson’s disease. *ACSL6* expression is down regulated in patients with AD and relevant models. In some experiments, *Acsl6*^−/−^ mice showed significantly impaired short-term spatial working memory. Hence, *ACSL6* promotes DHA enrichment in neurons. Down-regulation of *ACSL6* affects motor function, memory, and age-related neuroinflammation and increases the risk of AD.

Using JASPAR CORE 2020, we identified that the activity of the enhancer containing rs3776011 is mediated by the TF DMRTA2. Meanwhile, DMRTA2 was expressed in many human brain regions according to the Human Protein Atlas dataset. According to previous research [52], TF DMRTA2 efficiently elevates the enhancer activity. Therefore, the rs3776011 T allele was predicted to have increased binding affinity for DMRTA2 according to JASPAR, which may enhance enhancer–promoter interactions and is associated with increased *ACSL6* expression. In contrast, the rs3776011-C allele does not bind DMRTA2, which represses enhancer activity, resulting in relatively lower *ACSL6* expression.

However, these sequence-based motif predictions do not demonstrate allele-specific DMRTA2 occupancy in vivo, and the present study did not directly measure allele-dependent enhancer activity or genotype-dependent chromatin contact strength. The relatively low expression level of *ACSL6* has been associated with increased AD risk. Taken together, the combined motif, chromatin-interaction, and eQTL observations are interpreted as a testable regulatory hypothesis in which sequence variation at the rs3776011-containing regulatory region may influence *ACSL6* expression via altered DMRTA2 binding. Experimental assays will be required to determine whether DMRTA2 directly participates in this regulatory relationship.

## 3. Discussion

Regulatory DNA elements, including enhancers and PIRs, govern gene expression via physical interactions with target gene promoters. These functional contacts are frequently established through long-range chromosomal interactions that span extensive genomic distances. Three-dimensional chromatin structure datasets, including Hi-C and PCHi-C profiles, enable the detection of significant genomic interactions, which in turn allow putative regulatory sequences to be mapped to their corresponding target genes across both promoter and distal genomic regions. In the present study, hundreds of thousands of long-range cis-interactions between promoters and SNPs residing within distal regulatory elements (such as enhancers and PIRs) were identified. Hi-C and PCHi-C data from the brain tissues, hippocampus and brain cortex, were utilized to link distal promoter-interacting regions to their target genes dysregulated in AD.

The contribution of the present study lies primarily in the systematic functional interpretation of AD-associated variants using complementary brain-specific 3D chromatin information rather than in the development of a new statistical SNP-to-gene algorithm. By combining Hi-C-derived enhancer–promoter relationships with PCHi-C-derived promoter-centered interactions, the analysis provides two complementary routes for linking distal non-coding variants to candidate regulatory target genes. This design is intended to refine functional interpretation and generate experimentally testable regulatory hypotheses rather than to establish variant-level or gene-level causality.

The genes closest to the SNP are not necessarily its target genes. We found that SNP located in the intron region of *GAB2* was not closely associated with the gene expression of GAB (*p*-value= $1.11\times 10^{-2}$ in brain cortex, Figure 4c). In addition, although rs3776011 was located in RAPGEF6, the eQTL of RAPGEF6 and rs3776011 (*p*-value = $6.31\times 10^{-5}$ and *p*-value = $3.78\times 10^{-2}$ in brain cortex and hippocampus, respectively) was less significant compared to that of *ACSL6* and rs3776011 (*p*-value = $4.03\times 10^{-9}$ and *p*-value = $5.19\times 10^{-3}$ in brain cortex and hippocampus, respectively). Prior research by Brodie et al. showed that genes associated with or potentially affected by genetic variants can be located up to 2 megabase pairs (Mb) away from the corresponding SNPs [5]. Previous studies have demonstrated the important role of 3D chromatin architecture in the functional annotation of non-coding SNPs, and the analytical approach used in this study provides a systematic means of identifying putative target genes for these variants.

For the rs3776011–*ACSL6* locus specifically, variants across the broader 5q31.1 region and the functional roles of *ACSL6* in brain lipid metabolism and neuronal function have been reported in previous AD genetic and biological studies. The novelty of our analysis at this locus lies in integrating brain 3D chromatin contacts, expression association evidence and sequence-based motif predictions to nominate a distal regulatory relationship between the rs3776011-containing enhancer and *ACSL6*, rather than identifying a novel AD-associated locus or establishing a definitive causal mechanism.

The present analysis should also be considered in the context of established post-GWAS mapping approaches. H-MAGMA incorporates brain chromatin contacts into gene-level association analysis, whereas FUMA, INFERNO, and related integrative approaches combine positional, eQTL, and functional regulatory evidence. ABC-based approaches use cell-type-specific regulatory activity and chromatin contact to predict enhancer–gene relationships. In AD, Lutz et al. [26] integrated brain chromatin-state annotations with enhancer–promoter interaction information to narrow candidate genes at selected LOAD loci. Our analysis is complementary to these approaches: rather than calculating a new gene-level association statistic or calibrated SNP-to-gene score, it traces candidate regulatory relationships from AD-associated variants located in enhancers or PIRs to interacting promoters using two complementary brain 3D chromatin datasets.

In this study, we performed functional annotation of AD-associated variants located in enhancer regions and promoter-interacting regions (PIRs), resulting in the identification of 608 unique candidate regulatory target genes supported by chromatin-interaction evidence. We showed that SNP in PIRs is highly enriched for enhancer regions (*p*-value = $6.214\times 10^{-19}$, hypergeometric test). This result is consistent with previous studies that PIRs are highly enriched for active chromatin features (including enhancers, open chromatin features and so on) [8].

Comparison with the ABC model and the AD-specific strategy of Lutz et al. [26] showed that the different approaches generated partially overlapping but non-identical SNP–gene assignments (Figure S1 of Supplementary Materials S5). Their GTEx nominal eQTL P-value distributions also differed across brain cortex and hippocampus. These findings suggest that the different mapping strategies capture complementary aspects of regulatory architecture. Because eQTL association is not a gold-standard measure of causal SNP–gene mapping, this comparison should be interpreted as an assessment of independent expression-associated support rather than proof of universal superiority of any single method.

Among the modules identified in crosstalk network, “immune module” as the first module and “neural module” as the second module, are an unexpected match with Hu et al.’s analysis according to pathway crosstalk analysis on AD-related genes [53]. Meanwhile, it is interesting that these two modules are consistent with our previous findings [54]. Hence, we speculate that AD is caused by multiple mechanisms, and inflammatory response and dysregulated neuronal signaling-related pathways are two main etiological factors to promote AD.

However, there remain limitations with respect to our strategy for functional annotation of AD-associated SNPs based on 3D chromatin structure. Firstly, inevitably, the relatively modest amount of available brain chromatin interaction data restricted the statistical power to identify long-range cis-interactions. In addition, the 40 kb resolution of the Hi-C data limits the precision of localizing individual chromatin interactions, and the identified SNP–gene relationships should therefore be interpreted at the locus level rather than as nucleotide-level contacts. An additional limitation is that the study was based on reported GWAS Catalog lead variants and their strong LD proxies rather than complete locus-level GWAS summary statistics. Consequently, the present analysis does not statistically resolve causal variants within individual loci. Formal statistical fine-mapping requires comprehensive locus-level association statistics together with an appropriate ancestry-matched LD reference to estimate variant-level posterior probabilities of causality, whereas formal GWAS–eQTL colocalization requires compatible regional summary statistics from both the disease GWAS and the molecular QTL dataset to evaluate whether the two association signals are likely to share a causal variant. These requirements are beyond the data structure of the present study. Therefore, LD expansion in our analysis was used to define a candidate regulatory search space rather than to identify causal variants, and GTEx eQTL associations were treated as complementary expression-associated evidence rather than formal colocalization evidence. The resulting SNP–gene relationships should consequently be interpreted as chromatin-contact-supported regulatory candidates rather than fine-mapped causal variant–gene relationships. Future studies based on homogeneous full GWAS summary statistics could integrate statistical fine-mapping and formal colocalization with the present 3D chromatin-based annotation strategy.

Secondly, while studying chromatin interactions in healthy brain tissues enables the detection of regulatory interactions in the absence of dysregulation, the epigenomic characterization of AD patient-derived cells will be important to glean specific insights into how the 3D epigenome is altered in disease. Additional experiments are necessary to determine 3D genomic interactions in the brain tissues of AD patients. Thirdly, SNPs and their candidate effector genes are identified through bioinformatics analyses in this strategy, and the relevant experiments in vitro and vivo model systems are lacking. Fourth, although our functional annotation strategy can identify the majority of AD-associated lead SNPs and their target genes (552/787), there are still nearly 30% SNPs that have not been effectively annotated.

In the future, the 3D epigenomes of more brain cells and tissues from healthy individuals and AD patients will be sequencing. The integration of omics big data and bioinformatics in a systems biology perspective will resulting in an improved understanding of the underlying disease molecular mechanisms. Meanwhile, application of CRISPR technology could be employed to prove the interactions of SNPs and their candidate effector genes, it may lead to a better understanding of SNP impact on gene function, health of an individual and disease progression.

## 4. Materials and Methods

### 4.1. Identification and Annotation of AD-Associated SNPs

AD lead SNPs were obtained from the GWAS Catalog database (https://www.ebi.ac.uk/gwas/) (accessed on 1 August 2026) [2]. AD lead SNPs were selected according to the reported genome-wide association results. AD-associated lead variants were collected using a study-specific suggestive inclusion threshold of $p<1\times 10^{-6}$. This threshold was selected to retain a broader set of potentially relevant variants for downstream functional annotation rather than to define genome-wide significant or causal variants. Therefore, all variants included at this stage were treated as candidates requiring additional functional evidence. Variants were restricted to European ancestry, and records with “Alzheimer” in the “DISEASE/TRAIT” column were retained. The genomic positions of all SNPs were standardized to the human reference genome (hg19) based on their reference SNP IDs (rsID). SNPs without rsID were manually curated. A total of 787 unique lead SNPs associated with AD were collected for downstream analysis. The complete list of these variants is provided in Supplementary File S3.

To broaden the set of variants considered for downstream functional annotation, AD-associated lead SNPs were expanded based on linkage disequilibrium (LD) using HaploReg v4.1. database (http://compbio.mit.edu/HaploReg) (accessed on 1 August 2026) [55]. Variants in strong LD ($r^{2}\geq 0.8$) with the reported lead SNPs in European populations were retained.

This LD expansion was used to define a broader candidate regulatory search space for subsequent genomic and 3D chromatin annotation and was not intended to perform statistical fine-mapping or to identify causal variants. Because the lead variants were collected across multiple GWAS Catalog studies rather than from a single homogeneous GWAS summary-statistics dataset, the present analysis was designed for cross-study functional annotation rather than locus-level statistical fine-mapping or formal GWAS–eQTL colocalization. Accordingly, all LD-expanded variants are referred to throughout this study as candidate variants for functional annotation rather than fine-mapped or causal variants. After LD expansion and removal of duplicates, 6164 unique AD-associated variants were retained for downstream analysis.

The genomic characteristics of AD-associated variants were further annotated using ANNOVAR (version 2020Jun08). Genomic locations of the AD-associated variants were annotated using ANNOVAR based on the Func.refGene field. For visualization in Figure 2a, the detailed ANNOVAR annotations were consolidated into seven genomic-location groups: exonic, UTR5, UTR3, intronic, upstream, downstream, and intergenic. Specifically, UTR5 and ncRNA_UTR5 were combined as UTR5, UTR3 and ncRNA_UTR3 were combined as UTR3, and intronic and ncRNA_intronic were combined as intronic. The exonic, upstream, downstream, and intergenic categories were retained according to their corresponding ANNOVAR annotations. According to their genomic locations, SNPs were classified into different functional categories, including coding regions, intronic regions, untranslated regions, promoter regions, and intergenic regions. The resulting SNP dataset was subsequently integrated with three-dimensional chromatin interaction information to identify potential regulatory relationships between AD-associated variants and their target genes.

### 4.2. Three-Dimensional Chromatin Structure Information

Three-dimensional chromatin interaction information was obtained from publicly available Hi-C and promoter capture Hi-C (PCHi-C) datasets generated from human brain tissues. Hi-C and PCHi-C datasets were retrieved from the Gene Expression Omnibus (GEO) database under accession numbers GSE87112 and GSE86189, respectively [29,30].

The Hi-C dataset generated by Schmitt et al. [29] was used to characterize genome-wide chromatin interactions in human brain tissues, including cerebral cortex (GSM2322542) and hippocampus (GSM2322543). Raw Hi-C sequencing reads were processed using the HiC-Pro pipeline (version 2.11.4) [56]. In brief, the paired-end reads were first aligned to the hg19 reference genome assembly using the default settings in BWA [57]. Unmapped sequencing reads were subjected to filtering, while putative PCR duplicates were eliminated using the Picard toolkit (https://broadinstitute.github.io/picard/) (accessed on 1 August 2026). Hi-C reads aligned to the human genome assembly hg19 were adopted for subsequent analysis; this reference build was selected because many published datasets were mapped to hg19 and because extensive annotations of candidate functional non-coding sequences are available for this assembly. To generate contact maps at the corresponding resolution, the genome was partitioned into 40 kb bins, and interaction frequencies were calculated by enumerating the number of reads mapped to each bin pair [58]. Normalization was performed via the Iterative Correction and Eigenvector decomposition (ICE) procedure [59] to mitigate technical biases present in the Hi-C dataset.

The processed Hi-C interaction maps were subsequently used to identify enhancer–promoter interactions through the PSYCHIC framework for downstream assignment of SNP-associated candidate effector genes.

PCHi-C data generated by the Ren laboratory were used to identify promoter-centered chromatin interactions in human brain tissues [30]. The processed interaction data were obtained from GEO under accession number GSE86189. Interaction frequencies were normalized according to HindIII restriction fragment resolution, and significant long-range interactions were used for linking promoter-interacting regions (PIRs) with candidate effector genes.

### 4.3. Functional Annotation Strategy of AD-Associated SNPs

SNPs associated with AD were assigned to their candidate effector genes according to genomic location and three-dimensional chromatin interaction evidence. Variants located within promoter or exon regions were directly linked to the corresponding genes based on the GENCODE v19 gene annotation (https://www.gencodegenes.org/human/release_19.html) (accessed on 1 August 2026). In this study, promoter regions were defined as the genomic regions within 2 kb upstream of the transcription start site (TSS) of each gene isoform. Intronic and intergenic SNPs located in enhancer regions and PIRs were assigned to their candidate effector genes based on the Hi-C and PCHi-C data from brain tissues, respectively (Figure 1).

For noncoding SNPs located in regulatory regions, chromatin interaction information from human brain tissues was incorporated to identify potential candidate effector genes. SNPs located in enhancer regions were connected with candidate effector genes according to enhancer–promoter interactions inferred from Hi-C data using the PSYCHIC algorithm. Enhancer regions were defined based on chromatin states identified by ChromHMM [60] using epigenomic datasets from ten human brain tissues collected by the Roadmap Epigenomics Project (http://egg2.wustl.edu/roadmap/web_portal/) (accessed on 1 August 2026). The full set of ten brain tissues comprises the following: hippocampus middle, substantia nigra, anterior caudate, cingulate gyrus, inferior temporal lobe, angular gyrus, dorsolateral prefrontal cortex, germinal matrix, fetal brain female, and fetal brain male.

Based on 12 distinct chromatin marks (H3K4me1, H3K4me2, H3K4me3, H3K9ac, H3K27ac, H4K20me1, H3K79me2, H3K36me3, H3K9me3, H3K27me3, H2A.Z, and DNase I-hypersensitive sites), the chromatin state model implemented via ChromHMM partitions the human genome into 25 discrete functional states [61]. For this analysis, six states associated with enhancer function were selected to serve as enhancer annotations, specifically Active Enhancer 1 (EnhA1), Active Enhancer 2 (EnhA2), Active Enhancer Flank (EnhAF), Weak Enhancer 1 (EnhW1), Weak Enhancer 2 (EnhW2), and Primary H3K27ac possible Enhancer (EnhAc). The EnhA1, EnhA2, and EnhAF states are distinguished by elevated levels of H3K4me1 and H3K27ac, two histone modifications that represent canonical markers of enhancer activity. By contrast, the weak enhancer states EnhW1 and EnhW2 exhibit high H3K4me1 signal alongside reduced H3K27ac abundance. The EnhAc state, meanwhile, displays diminished H3K4me1 levels paired with increased H3K27ac signal.

For SNPs located in promoter-interacting regions (PIRs), candidate effector genes were determined using significant promoter-centered interactions obtained from PCHi-C datasets. Following the criteria described in the original PCHi-C study, interactions with p<0.01 were considered significant [30] and were used for linking PIR-associated SNPs with their potential candidate effector genes.

### 4.4. Pathway Crosstalk Network Construction

To investigate the biological functions of genes associated with significant AD-related SNPs, functional enrichment analysis was performed using ToppGene [62], a web-based platform for biological process annotation. Pathways showing false discovery rate (*FDR*) values below 0.05 were considered significantly enriched and were selected for subsequent pathway interaction analysis.

Further, we established crosstalk between pathways to investigate the interactions of the significant enrichment pathways [63]. Only significant pathways with *FDR* < 0.05 were kept for the following analysis. Additionally, pathways harboring fewer than five candidate genes were excluded from subsequent analysis, as an overly limited set of candidate genes per pathway may result in sparse or biased connectivity with other pathways.

The interaction strength between pathway pairs was evaluated by integrating the overlap coefficient (OC) and Jaccard coefficient (JC). The average value of these two measurements was used as the overlap significance score (Overlap_Sig) to quantify the degree of gene sharing between pathways.

$$Overlap\_Sig_{A,B}=\frac{OC_{A,B}+JC_{A,B}}{2}$$

$$OC_{A,B}=\frac{\left|A⋂B\right|}{\min(|A|,|B|)}$$

and

$$JC_{A,B}=\left|\frac{A⋂B}{A⋃B}\right|$$

where A and B are the list of genes in two of these significant pathways.

The pathway crosstalk network was constructed using Cytoscape (version 3.9.1), a software platform for visualization and analysis of biological networks [64]. Spectral clustering algorithm was applied to partition the significant pathways within the network into several major modules, wherein pathways within each module exhibit more frequent mutual interactions than those across different modules, and tend to be associated with identical or closely related biological themes.

### 4.5. Comparison with Alternative SNP-to-Gene Mapping Approaches

To compare the chromatin-based SNP–gene assignments with alternative regulatory mapping strategies, we applied the Activity-by-Contact (ABC) enhancer–gene maps and the previously published AD-specific annotation strategy of Lutz et al. [26]. ABC enhancer–gene predictions were harmonized to GRCh37/hg19, and positive enhancer–gene links with ABC scores ≥ 0.015 were retained. Because enhancer–gene regulation is cell-type specific, H1-derived neuronal progenitor cells were used as a brain-relevant ABC annotation. AD-associated variants overlapping ABC enhancer regions were assigned to the corresponding predicted target genes, and duplicate SNP–gene assignments were collapsed by retaining the highest ABC score. SNP–gene pairs generated by the three approaches were subsequently queried against GTEx v8 cis-eQTL results from brain cortex and hippocampus. Nominal eQTL P-values were grouped into predefined intervals, whereas SNP–gene pairs not evaluated in GTEx cis-eQTL data were excluded from the denominator.

### 4.6. GTEx eQTL Dataset

The Genotype-Tissue Expression (GTEx) project (https://gtexportal.org) [65] provides a comprehensive resource for investigating relationships between genetic variation and tissue-specific gene expression. In this study, Publicly available GTEx v8 single-tissue eQTL results were queried to assess whether AD-associated variants were associated with the expression levels of their chromatin-supported candidate target genes in brain cortex and hippocampus. The *p*-values reported in this study are nominal *p*-values for individual SNP–gene associations, as provided by GTEx single-tissue eQTL analyses. The GTEx datasets contained RNA sequencing and genotype information from 205 brain cortex samples and 165 hippocampus samples. The associations between AD-associated SNPs and expression levels of corresponding candidate effector genes were examined using the available tissue-specific eQTL information.

### 4.7. Analysis of the Effects of SNPs on Transcription Factor (TF) Binding Motifs

The effects of AD-associated SNPs on TF binding motifs were analyzed using JASPAR 2020 [66]. JASPAR represents the most comprehensive open-access repository of matrix-based nucleotide profiles that characterize the binding specificities of transcription factors (TFs) across diverse species. The JASPAR CORE database (JASPAR 2020; available at http://jaspar.genereg.net/) contains a curated, non-redundant collection of profiles derived from published sets of experimentally defined TF-binding sites in eukaryotes.

For each significantly associated AD SNP, a 51-bp DNA sequence centered on the variant position was extracted and submitted to the JASPAR CORE database. Human-specific TF binding models were selected for motif analysis. SNPs were considered potential regulatory variants when alternative alleles resulted in altered TF binding motifs, and motif matches with JASPAR scores ≥ 5 were retained for further analysis.

The presence of a TF binding motif within a given genomic region does not guarantee that the corresponding TF actually binds to that locus in cellular contexts, as the TF may not be actively expressed in human brain tissue. So, the brain atlas in The Human protein atlas (https://www.proteinatlas.org/humanproteome/brain) (accessed on 1 August 2026) was used to ensure TF protein expressed in human brain tissue [67]. Through the visualization and integration of compiled datasets, the Brain Atlas provides a detailed characterization of protein expression across the human brain. For each gene-tissue pair, the consensus normalized expression (NX) value is defined as the highest NX score derived from the three included transcriptomics datasets: GTEx, FANTOM5, and HPA. A threshold of 1 NX was uniformly applied as the detection limit for all tissues represented in the database.

## 5. Conclusions

In conclusion, this study provides an integrative framework for interpreting non-coding Alzheimer’s disease-associated variants by incorporating three-dimensional chromatin interactions with complementary functional genomic evidence. By integrating GWAS-derived variants, brain Hi-C and promoter capture Hi-C data, publicly available brain eQTL information, and transcription factor binding predictions, we identified a set of candidate regulatory target genes supported by brain chromatin-interaction and complementary functional genomic evidence that may not be captured through conventional nearest-gene annotation alone. The resulting candidate genes showed functional convergence on neural and immune-related biological processes relevant to AD, while detailed analyses of the rs2373115–*NARS2*, rs6656401–*CR1*, and rs3776011–*ACSL6* loci illustrated how long-range chromatin interactions can generate testable hypotheses linking genetic variation to gene regulation. These findings underscore the critical role of 3D genome architecture in refining post-GWAS interpretation and narrowing candidate targets for mechanistic follow-up.

Several limitations of this study should be acknowledged when interpreting the findings. First, the relatively limited availability and 40 kb resolution of brain 3D chromatin datasets restrict the precision of chromatin interaction localization, and all identified SNP–gene relationships should be interpreted at the locus level rather than at nucleotide resolution. Second, our analysis was constructed using published GWAS Catalog lead variants and their strong linkage disequilibrium proxies, rather than complete locus-level GWAS summary statistics. Accordingly, the study cannot support formal statistical fine-mapping or GWAS–eQTL colocalization, and all reported associations represent candidate regulatory relationships rather than definitive causal variant–gene links. Third, the chromatin interaction data were derived from healthy brain tissues, and therefore may not fully capture disease-specific alterations of 3D genome organization in AD. Finally, the candidate target genes and regulatory hypotheses identified in this work are based entirely on bioinformatic analyses, and require further validation through in vitro and in vivo experiments.

It is worth noting that the identified SNP–gene relationships should be regarded as regulatory candidates, and future studies incorporating AD-specific chromatin maps, cell-type-resolved regulatory data, and experimental perturbation will be necessary to establish their functional and disease-relevant roles.

## Figures and Tables

**Table 1:** Pathways enriched in candidate effector genes.

Pathway	Source	*p* Value	PBH Value	Genes Included in the Pathway
Neurotrophin signaling pathway	MSigDB BIOCARTA	$1.883\times 10^{-6}$	$2.169\times 10^{-3}$	*MAPKAPK2*, *PDPK1*, *SHC3*, *MAP3K3*, *PLCG2*, *IRS2*, *MAPK1*, *MAPK7*, *MAPK10*, *IRS1*, *NFKBIE*, *NGFR*, *NTRK3*, *GSK3B*, *IRAK4*, *CDC42*
Translocation of ZAP-70 to Immunological synapse	REACTOME	$3.136\times 10^{-6}$	$2.407\times 10^{-3}$	*HLA-DQA2*, *HLA-DQB1*, *HLA-DQB2*, *HLA-DRA*, *HLA-DRB1*, *HLA-DRB5*, *ZAP70*
Neurotrophin signaling pathway_KEGG	KEGG	$4.334\times 10^{-6}$	$2.495\times 10^{-3}$	*MAPKAPK2*, *PDPK1*, *SHC3*, *MAP3K3*, *PLCG2*, *MAPK1*, *MAPK7*, *MAPK10*, *IRS1*, *NF-KBIE*, *NGFR*, *NTRK3*, *GSK3B*, *IRAK4*, *CDC42*
The Role of Eosinophils in the Chemokine Network of Allergy	MSigDB BIOCARTA	$1.149\times 10^{-5}$	$4.953\times 10^{-3}$	*HLA-DRA*, *HLA-DRB1*, *HLA-DRB5*, *CSF2*, *IL3*
Th1 and Th2 cell differentiation	KEGG	$2.785\times 10^{-5}$	$7.715\times 10^{-3}$	*HLA-DQA2*, *HLA-DQB1*, *HLA-DRA*, *HLA-DRB1*, *HLA-DRB5*, *ZAP70*, *MAPK1*, *MAPK10*, *MAML1*, *JAK2*, *NFKBIE*, *MAF*
B Lymphocyte Cell Surface Molecules	MSigDB BIOCARTA	$4.608\times 10^{-5}$	$9.125\times 10^{-3}$	*HLA-DRA*, *HLA-DRB1*, *HLA-DRB5*, *ICAM1*, *CR1*
Phosphorylation of CD3 and TCR zeta chains	REACTOME	$9.586\times 10^{-5}$	$1.380\times 10^{-2}$	*HLA-DQA2*, *HLA-DQB1*, *HLA-DQB2*, *HLA-DRA*, *HLA-DRB1*, *HLA-DRB5*
Generation of second messenger molecules	REACTOME	$1.052\times 10^{-4}$	$1.425\times 10^{-2}$	*HLA-DQA2*, *HLA-DQB1*, *HLA-DQB2*, *HLA-DRA*, *HLA-DRB1*, *HLA-DRB5*, *ZAP70*
PD-1 signaling	REACTOME	$1.214\times 10^{-4}$	$1.553\times 10^{-2}$	*HLA-DQA2*, *HLA-DQB1*, *HLA-DQB2*, *HLA-DRA*, *HLA-DRB1*, *HLA-DRB5*
Cytokines and Inflammatory Response	MSigDB BIOCARTA	$2.313\times 10^{-4}$	$2.537\times 10^{-2}$	*HLA-DRA*, *HLA-DRB1*, *HLA-DRB5*, *CSF2*, *IL3*, *IL10*
Hematopoietic cell lineage	MSigDB BIOCARTA	$3.711\times 10^{-4}$	$3.716\times 10^{-2}$	*EPO*, *HLA-DRA*, *HLA-DRB1*, *HLA-DRB5*, *CR1*, *CSF2*, *IL3*, *IL6R*, *CD55*, *GP1BA*
Costimulation by the CD28 family	REACTOME	$4.296\times 10^{-4}$	$4.122\times 10^{-2}$	*HLA-DQA2*, *HLA-DQB1*, *HLA-DQB2*, *HLA-DRA*, *PDPK1*, *HLA-DRB1*, *HLA-DRB5*, *PRR5*, *CDC42*
Calcineurin-regulated NFAT- dependent transcription in lymphocytes	BIOCARTA	$4.489\times 10^{-4}$	$4.135\times 10^{-2}$	*SLC3A2*, *CSF2*, *IL3*, *CASP3*, *EGR1*, *EGR3*, *MAF*
Th17 cell differentiation	KEGG	$5.078\times 10^{-4}$	$4.498\times 10^{-2}$	*HLA-DQA2*, *HLA-DQB1*, *HLA-DRA*, *HLA-DRB1*, *HLA-DRB5*, *ZAP70*, *MAPK1*, *MAPK10*, *IL6R*, *JAK2*, *NFKBIE*
Neurotrophic factor-mediated Trk receptor signaling	Pathway Interaction Database	$1.709\times 10^{-3}$	$4.969\times 10^{-2}$	*SHC3*, *MAPK1*, *GAB2*, *SQSTM1*, *NGFR*, *NTRK3*, *CDC42*

## Data Availability

All datasets reported in this paper are available at the Gene Expression Omnibus with accession numbers GEO: GSE87112 and GSE86189. The source code is available on GitHub (https://github.com/wz-ahubio/functional-annotation-of-alzheimers-disease-associated-snps-based-on-3d-chromatin-structure.git) (accessed on 5 September 2026).

## Supplementary Materials

The following supporting information can be downloaded at: https://www.mdpi.com/article/10.3390/ijms27188396/s1.
