# Supplementary material for: Functional Annotation of Alzheimer’s Disease-Associated SNPs Based on Three-Dimensional Chromatin Structure
Source: Int J Mol Sci. 2026 Sep 20;27(18):8396. doi: 10.3390/ijms27188396 (PMC13607722; doi:10.3390/ijms27188396)
Supplement: Supplementary file 1 [file ijms-27-08396-s001.zip › Supplementary Materials S5.pdf]

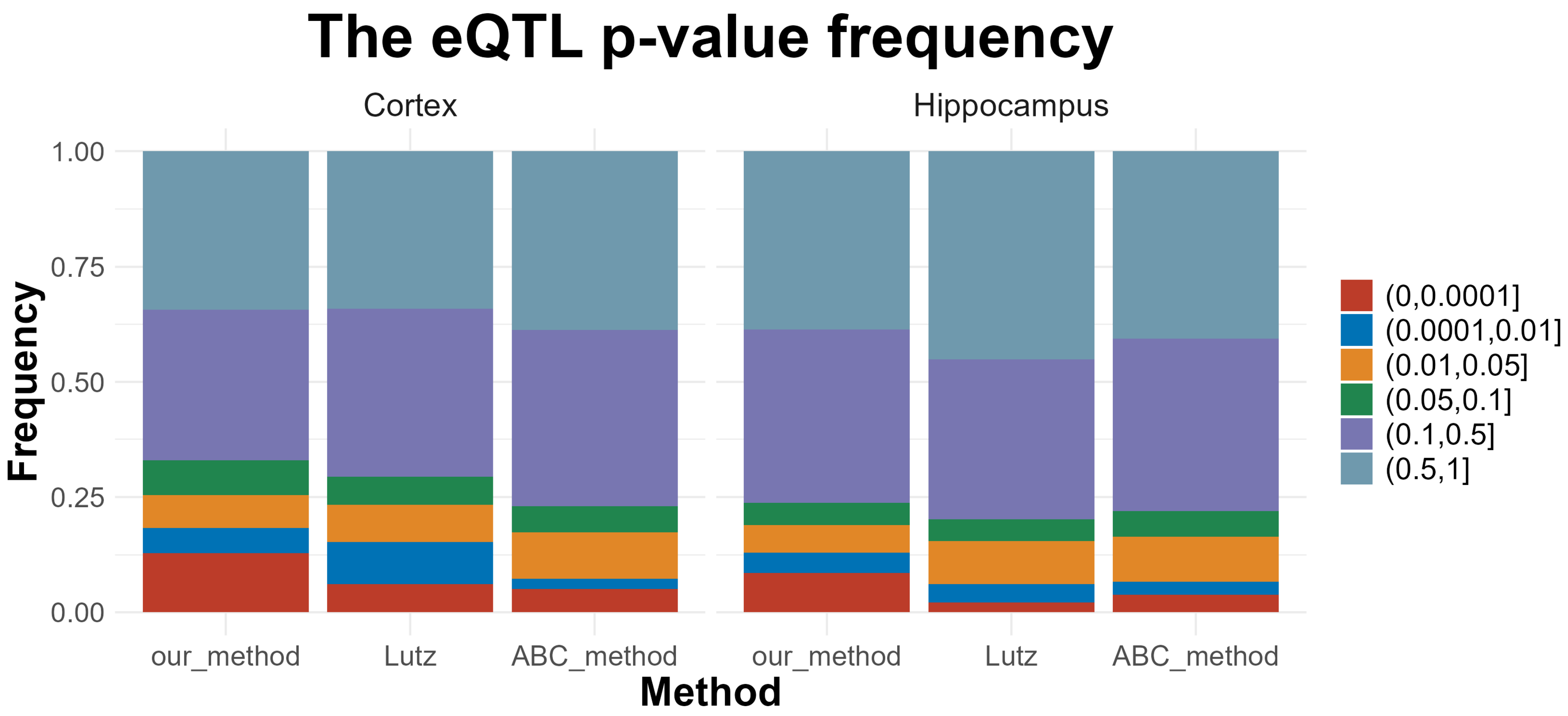

Figure S1. Comparison of eQTL P-value frequency distributions across three methods. Frequency distributions of GTEx nominal eQTL P-values for SNP–gene pairs from our method, the Lutz et al. strategy and the ABC model, shown separately for brain cortex and hippocampus. P-value bins are indicated in the legend.

Table S1. Genomic Functional Annotation of 13 AD-Associated Variants at the ACSL6 Locus.

**Target Gene**	**refsnp_id**	**chr_name**	**Location**	**Ref**	**Alt**	**Func.refGene**	**Gene.refGene**	**GeneDetail.refGene**	**cytoBand**
ACSL6	rs3776011	5	130868840	C	T	intronic	RAPGEF6	.	5q31.1
	rs380182	5	131319036	A	G	intronic	ACSL6	.	5q31.1
	rs6859469	5	130962546	C	T	intronic	RAPGEF6	.	5q31.1
	rs7706785	5	130954860	T	C	intronic	RAPGEF6	.	5q31.1
	rs3756290	5	130951750	A	G	intronic	RAPGEF6	.	5q31.1
	rs10040578	5	130950232	T	C	intronic	RAPGEF6	.	5q31.1
	rs27421	5	130743771	C	T	intergenic	CDC42SE2;RAPGEF6	dist=13389;dist=15843	5q31.1
	rs25831	5	130742849	C	A	intergenic	CDC42SE2;	dist=12467;dist=16765	5q31.1
	rs683749	5	131283376	C	T	intergenic	MEIKIN;ACSL6	dist=2020;dist=2291	5q31.1
	rs13170176	5	131138736	G	C	intergenic	FNIP1;MEIKIN	dist=6026;dist=3948	5q31.1
	rs1500114	5	131138120	C	A	intergenic	FNIP1;MEIKIN	dist=5410;dist=4564	5q31.1
	rs11956351	5	131137224	G	A	intergenic	FNIP1;MEIKIN	dist=4514;dist=5460	5q31.1
	rs2159243	5	131133900	G	A	intergenic	FNIP1;MEIKIN	dist=1190;dist=8784	5q31.1

Table S2. Genomic Coordinates of 6 Candidate Target Genes at the ACSL6 Locus.

SNP	Gene.Symbol	gene_id	Location	Length
rs3776011	ACSL6	ENSG00000164398	chr5:131,285,666-131,347,761	62,096 bp
	RAPGEF6	ENSG00000158987	chr5:130,759,614-130,970,922	211,309 bp
	FNIP1	ENSG00000217128	chr5:130,977,407-131,132,710	155,304 bp
	MEIKIN	ENSG00000239642	chr5:131,142,683-131,281,356	138,674 bp
	IL3	ENSG00000164399	chr5:131,396,348-131,398,897	2,550 bp
	CTC-432M15.3	ENSG00000273217	chr5:130,761,584-131,132,676	371,093 bp
